# Characterization of untranslated regions of the salmonid alphavirus 3 (SAV3) genome and construction of a SAV3 based replicon

**DOI:** 10.1186/1743-422X-6-173

**Published:** 2009-10-27

**Authors:** Marius Karlsen, Stephane Villoing, Espen Rimstad, Are Nylund

**Affiliations:** 1 Department of Biology, University of Bergen, Thor Møhlens gate 55, 5020 Bergen, Norway; 2 Intervet Norbio, Thor Møhlens gate 55, 5008 Bergen, Norway; 3 Norwegian School of Veterinary Science, Oslo, Norway

## Abstract

Salmonid alphavirus (SAV) causes disease in farmed salmonid fish and is divided into different genetic subtypes (SAV1-6). Here we report the cloning and characterization of the 5'- and 3'- untranslated regions (UTR) of a SAV3 isolated from Atlantic salmon in Norway. The sequences of the UTRs are very similar to those of SAV1 and SAV2, but single nucleotide polymorphisms are present, also in the 3' - conserved sequence element (3'-CSE). Prediction of the RNA secondary structure suggested putative stem-loop structures in both the 5'- and 3'-ends, similar to those of alphaviruses from the terrestrial environment, indicating that the general genome replication initiation strategy for alphaviruses is also utilized by SAV. A DNA replicon vector, pmSAV3, based upon a pVAX1 backbone and the SAV3 genome was constructed, and the SAV3 non-structural proteins were used to express a reporter gene controlled by the SAV3 subgenomic promoter. Transfection of pmSAV3 into CHSE and BF2 cell lines resulted in expression of the reporter protein, confirming that the cloned SAV3 replication apparatus and UTRs are functional in fish cells.

## Findings

Salmonid alphaviruses (SAVs) cause disease in farmed salmonids both in freshwater and the marine environment in Europe [1]. The virus, also known as *Salmon pancreas disease virus*, was molecularly characterized during the late 1990-ies, and assigned to the genus *Alphavirus *in the family *Togaviridae *[2,3]. Alphaviruses have single-stranded RNA genomes of 11-12 kb length with a 5'-terminal cap and a 3'-terminal polyadenylated tail. The coding sequences are organized into two large non-overlapping open reading frames (ORFs) that are flanked by three untranslated regions (UTRs). The first ORF is approximately 8 kb and encodes the non-structural proteins (nsPs) 1-4, while the second ORF is approximately 4 kb and encodes the structural proteins capsid, E3, E2, 6K, TF and E1. The second ORF is transcribed from an anti-sense genome under the control of a subgenomic promoter in the untranslated region that separates the two ORFs [4,5]. The genome is replicated by the nsPs, which together with host proteins make up the replicase complex (RC). The nsPs are translated as a polyprotein, P1234, that is cleaved by a papain-like serine protease of the nsP2 component. The different cleavage products of the RC have several roles during replication that include (i) recognition of viral genomic RNA and transcription of an anti-sense genome, (ii) recognition of the anti-sense genome and transcription of a new genome strand and (iii) recognition of the subgenomic promoter on the anti-sense genome and transcription of a subgenomic mRNA that contains the second ORF. The untranslated regions (UTRs) in the genomic 5'- and 3'- ends act as promoters for transcription of genomic and anti-genomic RNA. The RNA secondary structure found in conserved sequence elements (CSEs), rather than the primary sequence, appears to be the prominent factor in the function of these promoters [6].

Alphaviruses have been widely used in reverse genetics and protein expression systems. A common strategy used in alphaviral reverse genetics has been cloning of the viral genome under the control of an RNA polymerase promoter following transcription into a capped and polyadenylated self-replicating RNA [7]. In alphavirus-based replicons the subgenomic, second ORF is replaced with that of the gene of interest (GOI). Expression of the GOI is then executed by the alphavirus replication apparatus. Such replicons are frequently used for basic studies of alphavirus replication and for *in vivo *expression of GOIs, and can be used as vector systems in vaccination. Alphaviral based expression systems are useful for the latter application since they typically provide high expression of the transgene as well as activation of innate antiviral response in the transfected/transducted cell [8].

SAV is not genetically homogenous in Europe. Sequence comparisons of SAV isolates suggest that at least six distinct virus reservoirs exist and this has resulted in evolution into the subtypes SAV1-6 [9-11]. The coding sequence of nsP3 is particularly variable between the subtypes and contains several insertions/deletions with unknown effect in the C-terminal region. The SAV2 subtype appears to be widespread in freshwater farmed rainbow trout in continental Europe, whereas subtypes 1, 4, 5 and 6 have been found in Atlantic salmon from overlapping areas off the coast of Ireland, Northern Ireland and Scotland. In Norway a genetically homogeneous subtype, SAV3, is found to infect both Atlantic salmon and rainbow trout on the southwest coast, but has only occasionally been found in northern Norway [10,12].

**Figure 1:**
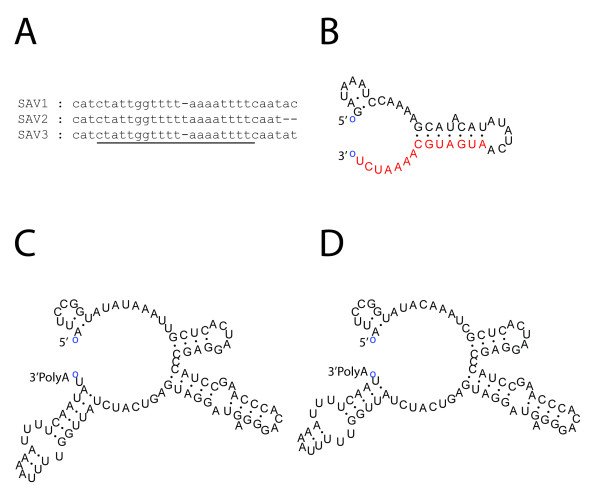
**Cloning and characterization of SAV3 5'- and 3'-ends**. The 5'- and 3'-ends of SAV3 were cloned and sequenced from the isolate SAVH20/03 and aligned to the 5'- and 3'-UTRs of other SAVs. A) Alignment of the region in the 3'-UTR containing a sequence homologous to the alphavirus 19 nt CSE (underlined). B) *In silico *RNA secondary structure analysis using Mfold of SAV3 5'-UTR and partial coding sequence. Red letters indicate coding sequence. C) Predicted RNA secondary structures of the 3'-UTRs of SAV2 and SAV3. Position of the polyadenylated tail is indicated.

A replicon allowing viral subgenomic promoter-driven expression of a GOI, as well as a reverse genetics system, has been developed for an attenuated strain of SAV2 [13]. In that system the SAV2 genome was transcribed by either T7 RNA polymerase or cellular RNA polymerase II, and the system has been useful for functional studies of SAV2 [13]. SAV3 and SAV2 represent two subtypes of the Salmonid alphavirus species showing approximately 7.1% nucleotide sequence differences in their genomic sequences [10]. SAV3 causes disease in farmed salmonids in the marine grow-out phase, while SAV2 typically causes disease in rainbow trout fingerlings. The optimum temperature for replication may also differ, as it appears to be lower for SAV2 than for SAV3 [1]. In order to learn more about these differences, we sought to obtain tools to study the replication apparatus of SAV3. The genomic ends of SAV3 had not been characterized. Therefore, we cloned and sequenced these from chinook salmon embryo (CHSE) cell cultures infected with SAVH20/03 [10] passage 28, using 5'- and 3'- rapid amplification of cDNA ends (RACE) kits (Invitrogen) as recommended by the manufacturer. Nucleotide sequence alignment of SAV3 UTRs with those of SAV1 and SAV2 (sequences of the UTRs of SAV4-6 subtypes were not available) demonstrated 100% identity of the 5'-UTR of SAV3 (SAVH20/03) and SAV2 strain rSDV, while four nucleotide polymorphisms were present in the 3'-UTR. Interestingly, one of these polymorphisms was found in the 3'-CSE (Fig. 1a). The 3'-CSE is conserved among alphaviruses, and functions as promoter for the initiation of minus-strand transcription [6].

The 5'- and 3'-UTRs of SAV are the shortest known among alphaviruses [14]. This has caused speculation as to whether SAV transcription initiation could be independent of a stem-loop structure in the genomic 5'- end [13]. Since secondary structure rather than nucleotide sequence in the 5'-UTR is decisive for initiation of genomic replication in other alphaviruses [6], the lack of a stem-loop in the SAV 5'-UTR would imply a different strategy for polymerase/promoter recognition. The distinct phylogenetic position of SAV, and vast evolutionary distance from terrestrial alphaviruses could make this plausible [2,15]. However, *in silico *analysis using Mfold RNA secondary structure predictor [16] with folding temperature set to 14°C (the replication temperature for SAV3), suggested that SAV 5'-UTR might form two short stem-loops in the 5'-end, and that a portion of the coding sequence of nsP1 is likely to be part of the second structure (Fig. 1b). In the 3'-UTR, four stem loops were predicted (Fig. 1c). Due to the observed polymorphisms in the 3'-CSE, it is predicted that SAV3 has a slightly longer stem loop than SAV2 (Fig. 1d).

**Figure 2:**
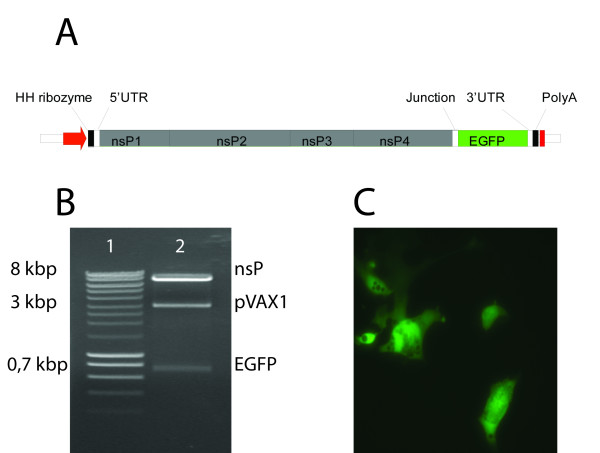
**Construction and evaluation of a SAV3 based replicon**. A) A SAV3 replicon is launched from the CMV promoter (red arrow) in the pVAX1 backbone, and transcription stops at the BGH polyA signal (red box). A synthetic DNA encoding a hammerhead ribozyme was fused to the SAV3 5'-UTR and a polyadenylated tail was fused to the 3'-UTR. The ORF encoding the SAV3 structural proteins was replaced by an ORF encoding EGFP inserted between introduced AgeI and AscI sites. Position of the EcoRI site used for restriction enzyme analysis is indicated. B) Restriction enzyme analysis by digestion of pmSAV3 with EcoRI, AgeI and AscI. Lane 1: Smartladder (Eurogentec). Lane 2: pmSAV3 after triple digest with EcoRI, AgeI and AscI. Bands corresponding to the pVAX1 backbone, nsP coding sequence and EGFP coding sequence are indicated. C) Expression of EGFP in BF2 cells after transfection with pmSAV3. Both CHSE and BF2 cells facilitated successful expression of the EGFP reporter. EGFP expression became visible from day 2 p.t. in CHSE cells and 3 d.p.t. in BF2 cells.

Knowledge of the ultimate ends and thus full-length sequence of SAV3 (isolate SAVH20/03), allowed construction of a SAV3 based replicon. Using a similar strategy as previously used for SAV2 [13], we constructed the plasmid pmSAV3 (Fig. 2a). The plasmid has the following characteristics: (i) a pVAX1 (Invitrogen) backbone optimized for DNA-vaccination, with a transcription unit controlled by the cytomegalovirus (CMV) immediate early promoter and a bovine growth hormone (BGH) polyadenylation signal, (ii) the SAV3 genome in which the structural ORF has been replaced with that of the enhanced green fluorescence protein (EGFP), flanked by AgeI and AscI restriction enzyme sites, (iii) a polyadenylated tail fused directly to the 3'-UTR of SAV3, and (iv) a hammerhead ribozyme fused directly to the SAV3 5'-UTR. The latter was included since a correct genomic 5'-end has been reported to be crucial to obtain efficient replication with SAV2 [9]. Ribozyme generation was done by annealing oligos 5HHribo2/3HHribo2 followed by polymerisation using Klenow fragment (TaKaRa), as earlier described [13]. Primers and restriction enzyme sites that were used for cloning purposes are listed in Table 1. The authenticity of the plasmid construction was verified by EcoRI, AgeI and AscI (New England Biolabs) digestion (Fig. 2b) and by sequencing as previously described [12]. This information indicated that eight substitutions were present in the RC coding region compared to the nucleotide sequence of passage 20 of the parental strain SAVH20/03 (Table 2). One of the substitutions, the R to C in the nsP3 region, was reported to be present in passage 3 of SAVH20/03 [10], suggesting that it could be part of a viral quasispecies.

**Table 1:** Primers used for construction of pmSAV3.

**Primer**	**Sequence 5'-3'**	**REN site**
KP1	CC**GAATTC**GTTAAATCCAAAAGCATACATATATCAATGATGC	EcoRI
KP2	CCCGGGGCGGCCCCAAGGTCGAGAACTGAGTTG	
KP3	CCCGGGAGGAGTGACCGACTACTGCGTGAAGAAG	
KP4	GG**TCTAGA**GTATGATGCAGAAAATATTAAGG	XbaI
nsP2SacIF	**GAGCTC**ATGACTGCGGCTGCC	SacI
nsP3HpaIR	**GTTAAC**CAAGACTTCCTCTTCGGC	HpaI
AscI3UTRF	**GGCGCGCC**ATTCCGGTATATAAA	AscI
AscIGFPR	**GGCGCGCC**TTACTTGTACAGCTCGTCCATGC	AscI
XbaIAgeIKGFPF	**TCTAGACCAACC**ACCGGTGCCACCATGGTGAGCAAG	XbaI, AgeI
5HHribo2	GGG**GAGCTCGCTAGC**TGGATTTATCCTGATGAGTCCGTGAGGACG AAACTATAGGAAAGGAATTCCTATAGTCGATAAATCCAAAAGC	SacI, NheI
3HHribo2	CCC**GCCGGC**GGAGGGGTTAGCTGTGAGATTTTGCATCATTGATATATG TATGCTTTTGGATTTATCGACTATAGGAATTCCTT	NaeI
NotIXbaIPolyAR	CC**GCGGCCGCTCTAGA**T_25_ATTGAAAATTTTAAAAACC	NotI, XbaI
NotIXbaIPolyA3R	CC**GCGGCCGCTCTAGA**T_23_ATATTGAAAATTTTAAAACC	NotI, XbaI

Restriction enzyme sites that were used during cloning are indicated in the sequence by bold letters.

**Table 2:** Mutations in the pmSAV3 coding region compared to the previously published sequence of SAVH20/03 passage 20 (Accession number DQ149204).

**Position in ORF**	**Protein product**	**Nucleotide substitution**	**Amino acid substitution**
306	nsP1	A to G	Silent
702	nsP1	T to C	Silent
2219	nsP2	C to A	A to D
4098	nsP2	A to G	Silent
5427	nsP3	C to T	Silent
5788	nsP3	T to C	R to C
7593	nsP4	A to G	Silent
7602	nsP4	A to G	Silent

Positions refer to nucleotide position in the ORF encoding SAV3 non-structural proteins.

Transfection of pmSAV3 into CHSE or BF2 cells using Amaxa nucleofector kit T (Lonza) or Metafectene Pro according to producer recommendations resulted in expression of the EGFP reporter that was visualized by fluorescence microscopy (Fig. 2c). In these experiments transfected cells were kept for 24 h at 20°C, then moved to 14°C, at which SAV3 replication is efficient [12]. The incubation step at 20°C enables the efficient transcription from the CMV promoter, which is more active at 20°C than at 14°C in CHSE cells (personal observation). In this SAV3 replicon system, expression of the EGFP reporter showed slower kinetics compared to the positive control used, i.e. EGFP under the direct control of the CMV promoter as in the plasmid pEGFP-N1 (Clontech), where EGFP typically can be observed as early as 6-12 h post transfection (p.t) (not shown). The earliest SAV3 replicon expression of EGFP was observed 2 days p.t. in CHSE cells and 3 days p.t. in BF2 cells. The number of positive cells peaked between days 6 and 8 p. t. in CHSE cells and around day 14 p.t. in BF2 cells.

In order to obtain efficient replication the polyadenylated tail had to be fused directly to the 3'-UTR of the SAV genome. Versions of pmSAV3 replicon constructs in which polyadenylation was initiated by the BGH signal in the vector backbone were not functional (not shown). This is similar to previous reports for DNA-launched SINV replicons [17], and suggests that SAV expression is sensitive for erroneous sequences between the 3'-UTR and the polyadenylated tail. Presumably, this has a negative effect on minus-strand synthesis as observed for SINV [18]. A different construct, pmSAV3M10, where the SAV3 3'-CSE was exchanged with SAV2 3'-CSE was also tested for its ability to express the reporter. This plasmid was generated by amplifying the 3'-UTR with primers AscI3UTRF and NotIXbaIPolyAR, where the latter primer sequence contained the SAV2 3'-CSE (Table 2). The 3'-UTR of pmSAV3 was then exchanged with the amplification product through AscI/XbaI cleavage and ligation. In transfection studies this construct showed expression kinetics similar to those observed for pmSAV3, confirming that the SAV3 replicase complex is able to recognise the SAV2 3'-CSE during replication. Moreover, this suggests that the polymorphisms observed in the 3'-CSE have little or no impact for SAV replication, and as predicted by *in silico *analysis, are of minor importance for the RNA secondary structure.

The pmSAV3 based plasmids can become valuable tools for functional studies of the SAV3 that currently is regarded as enzootic on the Norwegian west coast, but they may also be used for development of vectored vaccines for use in cold-water fish. It will be of interest to compare the performance of SAV3- and SAV2-based replicons [13] in such studies.

## Competing interests

The authors declare that they have no competing interests.

## Authors' contributions

MK planned the study, conducted laboratory and bioinformatical work, analysed results and wrote the manuscript. SV contributed to conception and experimental design, and critically revised the manuscript. ER contributed to the paper by helping in establishment of some of the laboratory methods used, discussion throughout the study and reading and contributing to the writing of the manuscript. AN contributed to the design of the project, discussion through the experimental period, and contributed to the writing of the manuscript.
